# Progression‐Associated Loss of SMARCB1/INI1 Expression in Mismatch Repair‐Deficient Glioblastoma

**DOI:** 10.1111/nan.70105

**Published:** 2026-11-06

**Authors:** Enrico Küllenberg, Nils Warneke, Markus Holling, Abigail K. Suwala, Christian Thomas, Martin Hasselblatt

**Affiliations:** ^1^ Institute of Neuropathology University Hospital Münster Münster Germany; ^2^ Department of Neurosurgery University Hospital Münster Münster Germany; ^3^ Department of Neuropathology, Institute of Pathology Heidelberg University Hospital Heidelberg Germany

**Keywords:** chromatin remodelling, glioblastoma, mismatch repair deficiency, SMARCB1, tumour evolution

## Abstract

- A mismatch repair‐deficient glioblastoma acquired a second *SMARCB1* mutation with complete loss of SMARCB1 protein expression at recurrence.
- Acquired SMARCB1 deficiency may represent a progression‐associated event in mismatch repair‐deficient glioblastoma and also constitute a diagnostic pitfall.

## Graphical abstract

A mismatch repair‐deficient glioblastoma acquired a second *SMARCB1* mutation with complete loss of SMARCB1 protein expression at recurrence. Acquired SMARCB1 deficiency may represent a progression‐associated event in mismatch repair‐deficient glioblastoma and constitute a diagnostic pitfall.

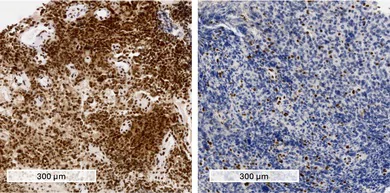

Mismatch repair‐deficient (dMMR) glioblastomas are rare hypermutated malignant gliomas characterized by treatment resistance and ongoing molecular evolution [1]. Alterations of the SWI/SNF chromatin‐remodelling complex, including SMARCB1 (also known as INI1), are a hallmark of atypical teratoid/rhabdoid tumour but are uncommon in malignant gliomas [2, 3, 4, 5]. Here, we document progression‐associated SMARCB1 deficiency in a molecularly characterized dMMR glioblastoma, representing, to our knowledge, the first report of acquired SMARCB1 loss in this context. Our findings suggest that defective DNA repair and altered chromatin remodelling may converge during tumour evolution and highlight acquired SMARCB1 loss as a potential diagnostic pitfall in recurrent malignant gliomas.

A 46‐year‐old man with no significant past medical history presented with visual deficits. There was no known family history suggestive of a hereditary cancer predisposition syndrome. Magnetic resonance imaging (MRI) revealed a ring‐enhancing mass in the left parieto‐occipital region, suggestive of a malignant glioma (Figure 1A). He underwent 5‐ALA‐guided gross‐total resection, with no residual contrast‐enhancing tumour evident on postoperative MRI. Histopathological examination showed a diffusely infiltrating astrocytic neoplasm with palisading necrosis and microvascular proliferation (Figure 1B). Immunohistochemical staining for mutant IDH1 (R132H) was negative, and the tumour was diagnosed as glioblastoma, IDH‐wildtype, WHO grade 4. Immunohistochemistry showed retained expression of MLH1, PMS2, and MSH2, with loss of MSH6 expression in the majority of tumour cells (Figure 1C), consistent with mismatch repair deficiency. Targeted next‐generation panel sequencing was performed with a custom targeted hybrid‐capture NGS panel (Twist Bioscience; version 4.1, total target size 170,642 bp) as recently described [6] on an Illumina NextSeq device. Reads were aligned to GRCh37 (Ensembl release 75), and variants were called within the panel target regions using DRAGEN (Illumina, v07.031.818.4.5.4) and annotated with Ensembl VEP v100. Sequencing confirmed the absence of *IDH1* and *IDH2* mutations and identified mutations in *PIK3CA*, *EGFR*, *PTEN*, *DICER1*, and *TP53*, as well as a single *SMARCB1* missense variant (p.R366C, VAF 51%), classified as likely oncogenic in OncoKB (https://www.oncokb.org/gene/SMARCB1/somatic/R366C). MSH6 was not covered by the targeted sequencing panel, precluding molecular characterization of the underlying MSH6 defect. Nuclear SMARCB1 protein expression was retained (Figure 1D). The MGMT promoter was unmethylated. The patient subsequently received standard focal radiotherapy with concomitant and adjuvant temozolomide together with tumour‐treating fields.

**FIGURE 1:**
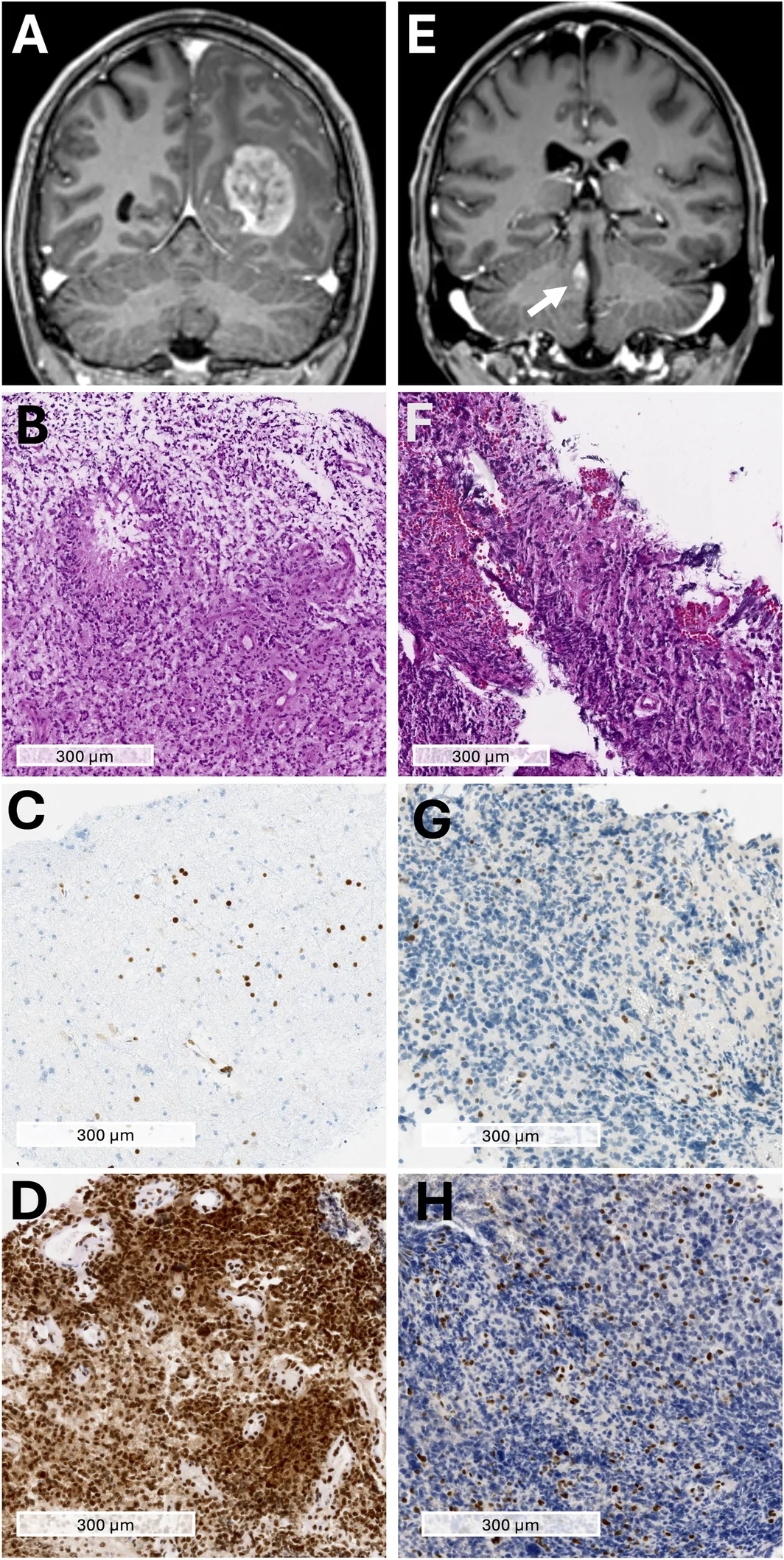
Radiological, histopathological and molecular evolution of a mismatch repair‐deficient glioblastoma. Left column, primary left parieto‐occipital tumour; right column, recurrent pontine tumour. (A) Contrast‐enhanced T1‐weighted MRI at initial diagnosis showing a ring‐enhancing left parieto‐occipital lesion. (B) Haematoxylin and eosin (H&E) staining demonstrating glioblastoma with palisading necrosis and microvascular proliferation. (C) MSH6 immunohistochemistry showing loss of nuclear expression in the majority of tumour cells. Note retained staining in nonneoplastic cells (internal positive control). (D) SMARCB1 immunohistochemistry showing retained nuclear expression. (E) Follow‐up contrast‐enhanced T1‐weighted MRI 14 months after initial diagnosis showing a distant pontine lesion consistent with tumour progression (arrow). (F) H&E staining of the recurrent tumour showing histopathological features similar to those of the primary lesion. (G) MSH6 immunohistochemistry showing persistent loss of nuclear expression in the majority of tumour cells. Note retained staining in nonneoplastic cells (internal positive control). (H) SMARCB1 immunohistochemistry showing acquired complete loss of nuclear expression in tumour cells. Note retained staining in nonneoplastic cells (internal positive control).

Fourteen months later, routine follow‐up MRI revealed a distant pontine lesion consistent with tumour progression (Figure 1E). Navigation‐ and fluorescence‐guided biopsy demonstrated a histopathologically similar glioblastoma (Figure 1F), and immunohistochemistry showed consistent loss of MSH6 expression in the majority of tumour cells (Figure 1G). Panel sequencing of the recurrent tumour confirmed the original *SMARCB1* missense mutation (p.R366C, VAF 46%). In addition, a *SMARCB1* missense mutation (p.R377C, VAF 43%), classified as likely oncogenic in OncoKB (https://www.oncokb.org/gene/SMARCB1/somatic/R377C) was identified. No reads supporting *SMARCB1* p.R377C were detected in the primary tumour despite high exon 9 coverage (mean 2464×; all bases ≥1000×), ruling out insufficient sequencing depth as an explanation. In contrast to the primary tumour, the recurrent lesion showed complete loss of nuclear SMARCB1 protein expression, consistent with acquired SMARCB1 deficiency (Figure 1H). DNA methylation profiling (Illumina Infinium MethylationEPIC BeadChip; DKFZ Brain Tumour Classifier v12.8) was performed on both samples. Both the primary and recurrent tumour independently classified as “adult‐type diffuse high‐grade glioma, IDH‐wildtype, subtype E” (calibrated scores 1.00 and 0.99, respectively). Copy‐number analyses derived from methylation array data demonstrated gains of chromosome 3p but no copy‐number alteration involving the *SMARCB1* locus on chromosome 22q in both tumours. Tumour mutational burden, calculated as the number of nonsynonymous variants per Mb of coding territory covered by the panel (53,018 bp across 32 genes) and (because no matched normal DNA was available) excluding variants listed in dbSNP or with a gnomAD popmax allele frequency ≥ 1% as putative germline variants, was estimated as 88.2 mutations/Mb in the primary tumour and 82.3 mutations/Mb in the recurrent tumour, consistent with a hypermutated phenotype. The patient died from progressive disease 3 months later while receiving palliative care.

To investigate whether *SMARCB1* alterations may occur in additional recurrent dMMR gliomas, we first searched our local archives but could not identify further cases with paired recurrent tissue available for analysis. We therefore extended our search to a recently characterized series of 34 primary dMMR gliomas [7]. Among the 34 patients, archived sequencing data from paired primary and recurrent tumours were available for one patient. This patient had been initially diagnosed with IDH‐wildtype glioblastoma at the age of 17 and experienced tumour recurrence 10 years later, at the age of 27. Interestingly, the recurrent tumour harboured a truncating *SMARCB1* mutation (p.Arg210*, VAF 26%) that had not been detected in the corresponding primary tumour. No residual tissue was available for SMARCB1/INI1 immunohistochemistry.

The presented case documents acquired SMARCB1 deficiency during progression of a primary dMMR glioblastoma. The primary tumour harboured a single *SMARCB1* missense variant with retained SMARCB1 protein expression, whereas the recurrent tumour acquired a second *SMARCB1* mutation accompanied by complete SMARCB1 protein loss. The acquisition of a second SMARCB1 variant accompanied by loss of SMARCB1/INI1 protein expression is consistent with progressive SMARCB1 inactivation, potentially involving biallelic disruption, although the allelic configuration of the two variants could not be determined.

Loss of SMARCB1 protein expression in a high‐grade CNS tumour classically raises the differential diagnosis of atypical teratoid/rhabdoid tumour [8]. In the present case, however, comparison with the primary tumour, characteristic glioblastoma morphology, and DNA methylation profiling supported recurrent glioblastoma and demonstrated that SMARCB1 loss represented an acquired progression‐associated phenotype. Thus, SMARCB1 deficiency should not invariably be regarded as a lineage‐defining event but may emerge secondarily during the evolution of malignant gliomas. Awareness of this possibility may help avoid diagnostic misclassification, particularly when only tissue from a recurrent tumour is available.

The dMMR phenotype in our case was already present in the primary tumour, as evidenced by loss of MSH6 expression and a markedly elevated tumour mutational burden prior to treatment. Thus, unlike the more common therapy‐associated dMMR observed in recurrent glioblastoma [1], the findings are consistent with a primary dMMR glioblastoma that subsequently acquired SMARCB1 deficiency during progression. There was no known family history suggestive of a hereditary cancer predisposition syndrome. Germline testing had not been performed, and no constitutional DNA was available for retrospective analysis; therefore, a hereditary mismatch repair syndrome could neither be confirmed nor excluded. Recent studies have identified primary dMMR IDH‐wildtype gliomas as a distinct epigenetic subgroup that includes adult‐type diffuse high‐grade glioma, IDH‐wildtype, subtype E [7]. The tumour in our case was likewise classified as subtype E, further supporting its classification as a primary dMMR glioblastoma. Whether acquired SMARCB1 deficiency contributed to tumour progression cannot be determined from a single case. Nevertheless, its emergence in a hypermutated dMMR tumour raises the possibility that genomic instability may facilitate acquisition of alterations affecting chromatin‐remodelling pathways. The scarcity of paired recurrent tissue limits assessment of how frequently SMARCB1 deficiency is acquired during progression of dMMR gliomas, particularly because surgical sampling at recurrence is uncommon. The identification of an additional tumour with an acquired truncating *SMARCB1* alteration suggests that such alterations may also occur in other dMMR gliomas, although neither protein loss nor functional consequences could be assessed in that case. Larger series of paired primary and recurrent tumours will be required to establish the frequency and biological significance of this phenomenon.

Taken together, our findings suggest that acquired SMARCB1 deficiency represents a previously unrecognized event in the evolution of dMMR glioblastoma. Its frequency, timing and biological significance remain to be established in larger molecularly characterized cohorts.

## Author Contributions

E.K. and M.Ha. drafted the manuscript. E.K., M.Ha. and C.T. performed histopathological and molecular studies. M.H. and N.W. contributed clinical data, and A.S. contributed to the identification and molecular analysis of an additional case. All co‐authors revised the manuscript critically for important intellectual content.

## Funding

The authors have nothing to report.

## Ethics Statement

The index patient gave informed consent for scientific use and publication of de‐identified clinical and molecular data. Publication of clinical and molecular data from the additional patient reported by Suwala et al. was covered by informed consent and approval from the Ethics Committee of Heidelberg University (no. S‐318/2022).

## Conflicts of Interest

The authors declare no conflicts of interest.

## Data Availability

The data that support the findings of this study are available from the corresponding author upon reasonable request.
